# RegenBase: a knowledge base of spinal cord injury biology for translational research

**DOI:** 10.1093/database/baw040

**Published:** 2016-04-06

**Authors:** Alison Callahan, Saminda W. Abeyruwan, Hassan Al-Ali, Kunie Sakurai, Adam R. Ferguson, Phillip G. Popovich, Nigam H. Shah, Ubbo Visser, John L. Bixby, Vance P. Lemmon

**Affiliations:** ^1^Stanford Center for Biomedical Informatics Research, Stanford University, Stanford, CA 94305; ^2^Department of Computer Science, University of Miami, Coral Gables, FL 33146; ^3^Miami Project to Cure Paralysis, University of Miami School of Medicine, Miami, FL 33136; ^4^Brain and Spinal Injury Center (BASIC), Department of Neurological Surgery, University of California, San Francisco; San Francisco Veterans Affairs Medical Center, San Francisco, CA 94143; ^5^Center for Brain and Spinal Cord Repair and the Department of Neuroscience, The Ohio State University, Columbus, OH 43210; ^6^Center for Computational Science, University of Miami, Coral Gables, FL 33146; ^7^Department of Cellular and Molecular Pharmacology, University of Miami School of Medicine, Miami, FL 33136, USA

## Abstract

Spinal cord injury (SCI) research is a data-rich field that aims to identify the biological mechanisms resulting in loss of function and mobility after SCI, as well as develop therapies that promote recovery after injury. SCI experimental methods, data and domain knowledge are locked in the largely unstructured text of scientific publications, making large scale integration with existing bioinformatics resources and subsequent analysis infeasible. The lack of standard reporting for experiment variables and results also makes experiment replicability a significant challenge. To address these challenges, we have developed RegenBase, a knowledge base of SCI biology. RegenBase integrates curated literature-sourced facts and experimental details, raw assay data profiling the effect of compounds on enzyme activity and cell growth, and structured SCI domain knowledge in the form of the first ontology for SCI, using Semantic Web representation languages and frameworks. RegenBase uses consistent identifier schemes and data representations that enable automated linking among RegenBase statements and also to other biological databases and electronic resources. By querying RegenBase, we have identified novel biological hypotheses linking the effects of perturbagens to observed behavioral outcomes after SCI. RegenBase is publicly available for browsing, querying and download.

**Database URL:** http://regenbase.org

## Introduction

Spinal cord injury (SCI) is a cause of significant disability and loss of quality of life. SCI research is an interdisciplinary field concerned with the biological mechanisms underlying loss of function and mobility after SCI and the discovery of therapies to promote the regeneration and repair of damaged neural tissue for functional recovery (1–6). Rat and mouse models are most commonly used to study the effects of SCI and responses to experimental treatments; a number of standard injury types and functional recovery measures have been developed for this purpose. Despite these advances, experiment replicability remains a significant challenge, with recent studies reporting that less than half of major experimental results are reproducible in a second laboratory (7, 8).

One reason for this problematic finding is inconsistent reporting of experimental methods and findings in published literature pertaining to SCI. To address this problem, we recently developed a minimum information guideline for the SCI research community—Minimum Information about a Spinal Cord Injury experiment (MIASCI) (9)—to encourage consistent reporting of all experimental details of a study. MIASCI includes fields for describing the model organisms used in a study including their genotype and phenotype, age, strain and housing environment, as well as any surgical variables including anesthetics used, injury type and severity, instrument used to administer injury, and post-surgical care. It also allows scientists to describe any perturbagens, cell transplants, or biomaterials used. Data fields for histology, immunochemistry and behavioral observation methods used to assess outcome measures in the study are also defined. Finally, MIASCI allows any scientist reporting a study to describe the primary findings in the form of simple structured assertions consisting of the agent(s) investigated (e.g. a perturbagen or injury type), their target(s) (e.g. axon growth, behavioral outcomes) and their effect(s).

Another significant barrier to discovery in SCI research is the lack of comprehensive, large-scale and widely accessible electronic resources, similar to the National Center for Biotechnology Information’s (NCBI) Gene Expression Omnibus for gene expression data (10), that capture experimental results from SCI studies and make them available for subsequent analysis by researchers. The availability of such resources in other areas of biological research has proven invaluable for driving new discoveries that were possible only through analyzing large volumes of data aggregated from significant numbers of experiments. This has yet to be achieved for neuroscience (11) and specifically in SCI research, though recent efforts have demonstrated the promise of large-scale integrated data resources in this domain (12). The majority of SCI research findings remains locked in publications that are human readable but not available for computational analysis, and are thus a valuable but largely untapped resource for informatics driven discovery.

Computational approaches for structuring and integrating biological data are an integral aspect of modern life sciences research. The breadth and depth of the literature and the volume of data generated by high throughput experimental methods makes it infeasible for experimental biologists to manually integrate these data for their own research. The application of Semantic Web standards and representation methods (including ontologies and linked data) has emerged as a promising solution to this synthesis problem. Recent work has demonstrated that structuring biological data using a linked data approach enables biological discoveries through data integration and the use of query languages (13–15), whereas the development and use of ontologies enables formal logic-based reasoning over biological and medical domain knowledge (16–18). Our recent work has also demonstrated the utility of a Semantic Web framework for data-driven hypothesis evaluation (19, 25) to discover novel aging-related genes in the model organism *Caenorhabditis* *elegans* (20).

We envision a scenario in which an SCI biologist can simultaneously interrogate relevant available literature, biological databases and experimental data to, for example, identify chemical compounds that improve behavioral outcomes after SCI in model organisms, and inhibit regulatory kinases *in vitro*, as well as identify the kinase targets of these compounds. With this new knowledge, she is able to hypothesize a possible mechanism by which these compounds affect behavior after injury and design experiments to test the hypothesis. After she completes these experiments, the data are available to other researchers who can use these results to design additional experiments (perhaps in another organism) and make new discoveries. Eventually, the increase in the pace of such findings can lead to the development of a therapy that is tested in a human clinical trial. We have developed RegenBase to capture domain knowledge, literature-sourced findings, and experimental data related to SCI in an online, publicly available resource to enable such a scenario.

## Materials and methods

Creating RegenBase required four distinct but related activities: ontology development, literature curation, data representation, and data integration. We describe each of these activities below with illustrative examples. Our guiding motivation for each activity was to create a representation of experimental data in the domain of SCI research that allows querying and data analysis to formulate and answer biological questions.

## Ontology development

**Figure 1.**
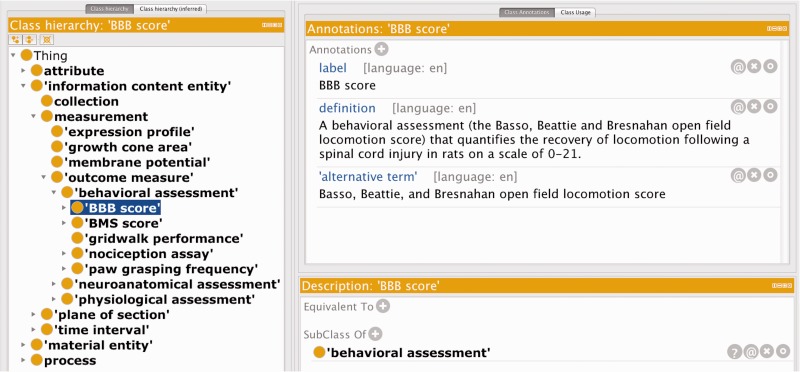
Subset of the new classes added to the RB, showing the human readable label, alternative term and definition for the BBB score class.

We created the RegenBase Ontology (RB) as a machine-understandable representation of the design and results of SCI experiments. We used a combination of automated and manual approaches for selecting entities to include. We first used a simple text processing pipeline to identify frequently mentioned 1–3 word entities from articles related to SCI research (retrieved by a keyword search of PubMed—the search terms used are listed in Supplementary Appendix S1). We manually reviewed the list of frequently mentioned entities to filter out those not specific to SCI biology. The remaining concepts became our preliminary set of classes. We worked with domain experts to develop the class hierarchy from our initial ‘seed’ classes and extend the ontology with classes representing aspects of experimental design (e.g. injury types, control groups, and treatment groups) and experimental outcomes (e.g. behavioral assays, measures of regeneration). For each class added, we determined its most appropriate placement in the RegenBase class hierarchy (adding parent classes as necessary to achieve a consistent granularity for all classes) and specified labels and synonyms as well as human readable definitions. A.C., S.W.A. and U.V. developed the class hierarchy, labels, and definitions by reviewing neuroscience and SCI literature and textbooks in close collaboration with domain experts V.P.L. and J.L.B., in an iterative process including extensive in-person discussions. We also consulted colleagues of V.P.L. and J.L.B. who have relevant expertise on specific classes, for both their correct placement in the RegenBase class hierarchy and their definitions. We used the Protégé ontology editor in combination with shared spreadsheets for class labels and definitions in all phases of ontology development. Figure 1 shows a subset of classes that were added to the RB, as well as an example of the labels, synonyms, and human readable definition for the Basso, Beattie and Bresnahan score class (‘BBB score’).

We also mapped RB classes to classes in other existing bio-ontologies whose labels have a lexical match. We first used the NCBO BioPortal Mappings web service to identify candidate class matches, and then restricted our set to those classes that were specific to the mapped ontology (i.e. not from an imported ontology) and where adding the mappings to the source ontology did not produce classification errors in the RB. The ontologies to which we currently map RegenBase classes are the Foundational Model of Anatomy and the Mammalian Phenotype Ontology (MP). We use the Simple Knowledge Organization Schema ‘is closely related to’ object property to assert RegenBase class mappings.

## Literature curation

Using the MIASCI reporting template (9), domain experts curated 42 published scientific articles describing experiments studying the effect of kinase inhibitors on recovery after SCI in rats and mice (Supplementary Appendix S2). In addition to capturing experimental details specified by MIASCI, our curators also captured primary findings from each article and represented these findings using bio-ontologies and terminologies.

Our curation workflow is a combination of manual and automated processes. Curators first manually capture experimental details and primary findings from each article, which are stored as spreadsheets with a predefined format (available at the RegenBase website). In this pilot phase, the primary findings were manually structured by author A.C. for addition to RegenBase from curator statements, and reviewed by authors V.P.L., J.L.B. and S.W.A. for correctness of content (including assignment of bio-ontology classes to entities and processes) and structure. An annotation tool developed by SWA then processed spreadsheets and structured primary findings corresponding to each article to create curated statements using classes from the RB as well as an additional set of bio-ontologies including the MP (21), Gene Ontology (GO) (22), BioAssay Ontology (BAO) (23) and Semanticscience Integrated Ontology (SIO) (24). These ontologies were used because they provide classes that match concepts frequently used by curators, including phenotypic outcomes (MP) and biological processes (GO) described in published experiments. As we curate a broader range of experiment types and publications, this set will grow to include additional bio-ontologies as needed.

Our custom annotation tool is designed to process each set of curated statements from a single article and generate a machine-understandable representation of these statements. The tool uses rules to process spreadsheets and produce an event-based representation in Resource Description Framework (RDF)/Web Ontology Language (OWL). The rules were developed to produce a consistent representation of literature-sourced relations between entities (both individuals and classes) as well as between entities and data (such as a string label or floating point number). This representation (described below) facilitates querying and aggregation across curated articles.

In addition to assigning types to entities using the above bio-ontologies, we achieved integration with existing data sources by associating small molecules curated from articles with their PubChem compound identifiers (CIDs), and by associating genes and proteins curated from articles with their NCBI Gene and UniProt identifiers, respectively. We used the UniProt Retrieve ID/Mapping and PubChem Power User Gateway services to perform identifier lookups by gene and protein names/official symbols, and chemical compound names or simplified molecular-input line-entry system strings, respectively. We used the MIRIAM registry and its identifiers.org service to specify all identifiers. Doing so enables integration with other data sources that also use this registry through the resolvable URIs it provides as identifiers, allowing users to retrieve additional structured information about small molecules, genes and proteins from their identifiers.org URIs. Our annotation tool also captures the provenance of all curated statements including the source publication and the curator of each statement. Annotations are thus curated reports of events, and are not interpreted as the ground truth of the event itself.

## Data representation

We designed an event-based representation for the curated literature-sourced primary findings describing SCI experiments and results. In this representation, experimental findings are captured as reported biological events measured by some experimental method. This representation is flexible (events may involve causative entities, dubbed ‘agents’, as well as the entities acted upon by an agent, dubbed ‘targets’, or both) and provides a consistent structure for a variety of experimental findings and their contexts (both physical and temporal). Context can include details such as the magnitude of experimentally observed effects (e.g. of agents affecting targets) and the timing of observed events relative to experimental setup. For example, SCI experiments often involve causing an injury to an animal model and measuring response to treatment at varying time points after injury or after application of a therapeutic treatment.

**Figure 2.**
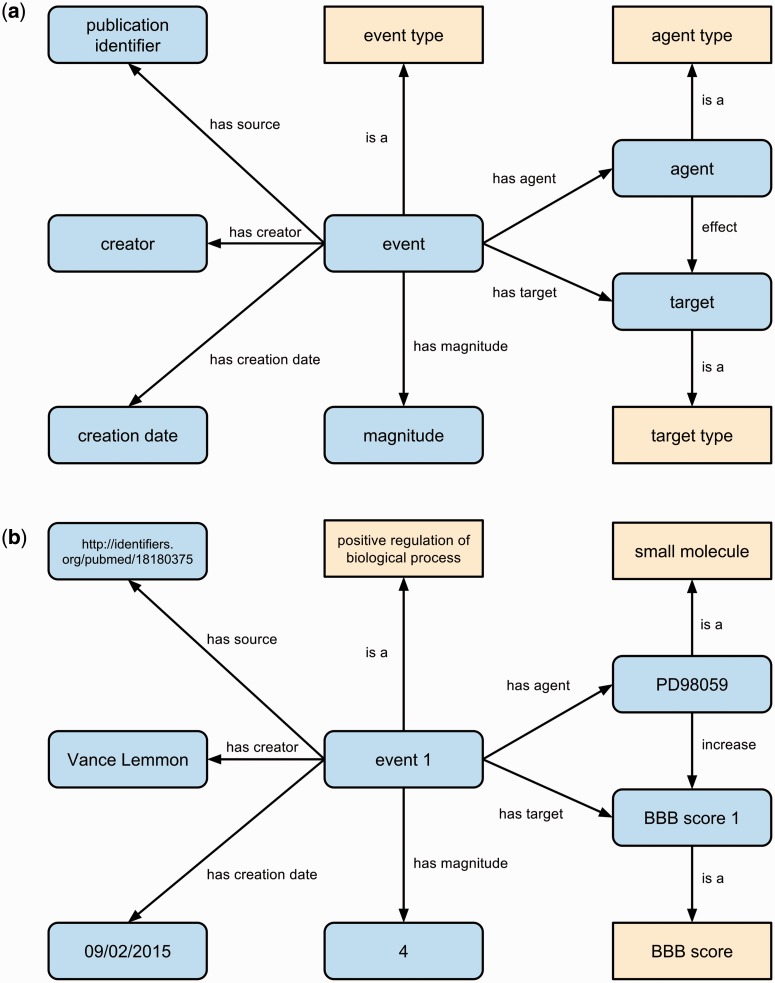
The generic event representation (**a**) for primary findings manually curated from the literature including provenance of the primary finding statement. Classes are shown as yellow boxes, individuals/literals as blue rounded boxes and relations between them as labeled arrows. An example event (**b**) in RegenBase specifies that the small molecule PD98059 was observed to increase BBB score by 4 points. Provenance for this event states that it was created by Vance Lemmon on September 9 2015 and was sourced from an article with PubMed identifier 18180375.

Our event representation specifies the type of event (using the bio-ontologies listed above), as well as the agent, target, effect, and magnitude of the observed effect of an agent on a target. The representation may optionally include a temporal context and a baseline relative to which an effect is observed. Each agent and target is also typed, and magnitude is quantified using data type properties and literal values. Figure 2 illustrates the generic event representation we use (Figure 2a) and an example from a curated article (Figure 2b). Any contextual details are linked to the event entity, and thus any number of additional contexts can be added without changing the underlying representation. We link all event statements to their provenance using the Dublin Core vocabulary, including the source article, as well as to information about the curation process, including the curator and date of curation.

In addition to literature-sourced curation, we populated RegenBase with a structured representation of experimental results from two kinds of assays:

- Kinase activity assays that quantify the activity of kinases exposed to different chemical perturbagens relative to a control.
- Assays that measure the effect of kinase inhibitors on neurite growth, a well-studied marker of recovery following SCI.

The kinase activity assay data profile the effect of perturbagens on the activities of kinases. Results from three assays are included in RegenBase: DiscoverRx’s KINOMEscan binding assay, NanoSyn’s fluorescence assay and Reaction Biology Corporation’s (RBC) radiometric assay. These assays measure kinase activity or binding in the presence of a perturbagen at a specified concentration with respect to a control. The KINOMEscan data consist of dissociation constant (*K*_d_) values for compound–kinase pairs, which we transformed to p*K*_d_ values by first converting the nanomolar *K*_d_ values to molar values, and then calculating the negative log 10 of each molar *K*_d_ value. The NanoSyn and RBC assay data consist of % inhibition values for compound–kinase pairs. In addition to the raw kinase assay data, we also include data from a derived activity measure (26) that integrates the results of multiple kinase assays into a single Kinase Inhibitor BioActivity (KIBA) value quantifying the effect of a given perturbagen on a kinase. We transformed KIBA values to the equivalent of a p*K*_i_ or p*K*_d_ and include the transformed data in RegenBase alongside the original values where they exist.

The neurite outgrowth assay data quantify the effects of compounds on neurite outgrowth with respect to a control, and are measured as the percentage of neurite total length observed relative to neurite total length when exposed to a control substance (dimethyl sulfoxide).

**Figure 3.**
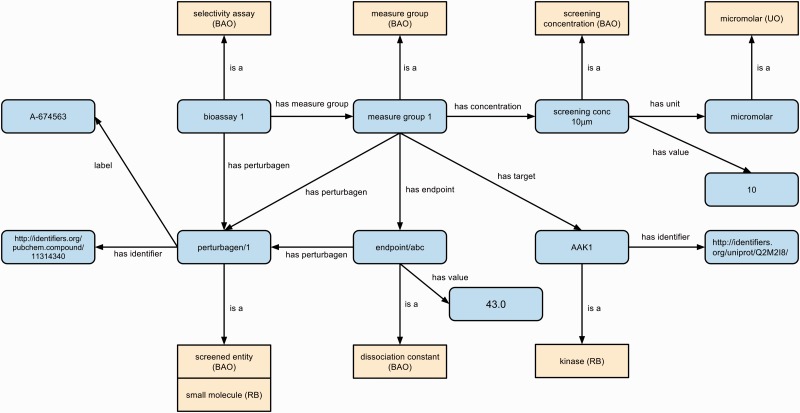
Example representation of assay experimental results using BAO, RB and UO. Classes are shown in yellow with their source ontology in brackets and instances are in blue. All relations except ‘label’ (RDFS) and ‘has identifier’ (SIO) are sourced from BAO. Classes are shown as yellow boxes (with the source ontology in brackets), individuals/literals as blue rounded boxes and relations between them as labeled arrows.

We used the BAO as well as the RB to represent assay details and experimental results. We used the NASA Units Ontology (UO) for all measurement units. We adopt BAO representation conventions (27) for grouping related assay results using the ‘Measure Group’ class, and use BAO classes and properties where possible. Figure 3 illustrates our assay representation approach with an example kinase activity assay.

We linked all assay perturbagens (screened entities) and kinase targets to their PubChem CIDs and UniProt protein identifiers where possible, to enable integration of existing publicly available data about these entities with RegenBase. For example, in Figure 3 we illustrate the results of a kinase activity assay measuring the effect of a 10 μM concentration of compound A674563 (PubChem CID 11314369) on the activity of adaptor-associated protein kinase 1 (human AAK1; UniProt ID Q2M2I8).

## Data encoding and integration

We used the RDF, a World Wide Web Consortium standard for linked data, to encode the literature-sourced event data and assay data that compose RegenBase. A.C. and S.W.A. developed custom programs in Java and Python for this purpose (available from the RegenBase website). We loaded these RDF data into an instance of the Virtuoso Open Source triplestore engine, version 7.10. RegenBase can thus be queried using the SPARQL Protocol and RDF Query Language (SPARQL) for fast data integration and retrieval, including queries that leverage basic subsumption reasoning and property paths, functionalities that are natively enabled by Virtuoso Open Source. The use of OWL ontologies as the backbone for organizing and structuring the RDF linked data in RegenBase also enables reasoning and consistency checking using a standalone reasoner such as Pellet (28) or Konclude (29).

Statements resulting from literature curation were manually reviewed by V.P.L., J.L.B. and A.C. for correctness and consistency. A subset of each assay dataset in RDF was also manually reviewed and compared with its source dataset (as provided by the original study investigators) for correctness. We reviewed RDF data using the Virtuoso Faceted Browser and SPARQL queries.

The use of publicly available identifiers for genes (from NCBI Gene), proteins (from UniProt) and chemicals (from PubChem) in RegenBase allows for automatic integration of data across publications from the literature annotations and across assay results. This enables the interrogation of RegenBase to quantify the effect of perturbagens on SCI recovery across multiple studies and assay types with simple queries, as we demonstrate in the following sections.

## Results

### RB and contents

The RB consists of 435 classes, 18 object properties and 8 data properties. The ontology is publicly available for search and download at NCBO’s BioPortal (see http://bioportal.bioontology.org/ontologies/RB).

The current version of RegenBase contains statements from 42 curated publications alongside the experimental results of kinase activity assays and compound screening assays for neurite outgrowth. Literature-sourced assertions contribute approximately 20 000 statements and assay experimental results contribute approximately 15 million statements.

The kinase assay data available in RegenBase capture the reported effects of nearly 52 000 compounds on the activity of 476 kinases, and the neurite outgrowth assay data capture the effect of 1606 compounds on neurite outgrowth in hippocampal neurons. We obtained kinase activity data from five sources: a published GlaxoSmithKline screen of kinase inhibition (30), a recent study of kinase inhibitor polypharmacology (31), two published studies of kinase inhibitor selectivity (32, 33) and one study that quantified kinase inhibitor selectivity by calculating an integrated activity measure from the results of multiple kinase inhibitor selectivity assays (26). The neurite outgrowth assay data are from two published studies on screening assays to identify drug targets that promote axon growth (31, 34).

The public RegenBase SPARQL endpoint and linked data files are available at http://regenbase.org. Both RegenBase and MIASCI are registered at biosharing.org.

### Querying RegenBase to identify potential targets for SCI drug therapies

RegenBase automatically links *in vivo* data, such as behavioral outcome measures associated with exposure to small molecules, to *in vitro* biochemical profiling data that describe potential targets of those small molecules, such as kinase inhibition profiles. From these links, researchers can develop hypotheses about molecular mechanisms for compounds that have observed behavioral effects.

By querying RegenBase, we have identified compounds that have been reported to improve locomotion after SCI (based on two open field outcome measures used in rodents, the BBB and BMS tests) and that also have been observed to strongly inhibit particular kinases *in vitro*. We have also identified the *in vitro* targets of these compounds, thereby offering insight into the mechanism(s) underlying the observed behavioral effects. We performed this task using a single query:

### QUERY 1—What are the kinase targets of perturbagens that have been observed to increase BBB/BMS score and inhibit kinase activity with either a % inhibition of at least 50% at a perturbagen concentration of 0.1 μM or a p*K*_d/_p*K*_i_ of at least 7?

This query retrieves all events where the agent is a small molecule and the target is a BBB or BMS score, where the source article reported that the agent increased BBB/BMS score. The agent must also have been observed to inhibit kinase activity relative to a control, in at least one assay measuring either percent inhibition (for which the threshold is ≥ 50%) at a perturbagen concentration of 0.1 μM or dissociation/inhibition constants (for which the threshold of the corresponding log-transformed values is ≥ 7). The query finds the identifiers of all compounds that satisfy these constraints and their kinase targets from each kinase activity assay, and returns the unique compound–kinase target pairs. The SPARQL query is shown in Supplementary Appendix S3; results are shown in Table 1.

**Table 1:** **.** Kinase targets that are strongly inhibited by compounds that have been independently observed to improve BBB score following injury

Perturbagen (with PubChem identifier)	Kinase targets strongly inhibited (with UniProt identifier)
SB 415286 (4210951)	GSK3B (P49841)
Fasudil (3547)	PRKX (P51817), ROCK2 (O75116), PRKACA (P17612)
PD 168393 (4708)	EGFR (P00533), ERBB2 (P04626), ERBB4 (Q15303)
SB 203580 (176155)	CSNK1A1 (P48729), CSNK1D (P48730), CSNK1E (P49674), GAK (O14976), JNK3 (P53779), MAPK11/p38-beta (Q15759), MAPK12 (P53778), MAPK13 (O15264), MAPK14/p38-alpha (Q16539), NLK (Q9UBE8), RIPK2 (O43353)

The first compound, SB 415286, inhibits GSK3 beta, a protein implicated in plasticity and sprouting. It has been reported that conditional knockout of GSK3 beta increases DRG axon regeneration and the sprouting of 5HT+ terminals after SCI (35). The result of this RegenBase query linking inhibition of GSK3B to a positive behavioral outcome in the BBB test is fully consistent, therefore, with other studies linking GSK3B to axon regeneration.

Fasudil is a known ROCK1/2 inhibitor, and its promotion of axon regeneration is normally ascribed to these activities. Fasudil has only modest effects on neurite growth for neurons on permissive substrates (34), but strongly promotes neurite growth on inhibitory substrates (36, 37).The biochemical profiling data queried by RegenBase reveal that Fasudil also inhibits PRKX, a protein recently identified as an important target for kinase inhibitors promoting axon growth (31). Thus, Fasudil’s growth promoting activity *in vitro*, and reported ability to improve behavioral outcomes *in vivo*, could both result from inhibiting PRKX together with ROCKs, and this ‘positive polypharmacology’ makes it an attractive candidate for treatment of SCI.

PD 168393 is an epidermal growth factor (EGF) receptor inhibitor and has been used in several SCI studies, including the NINDS SCI replication project (38–43). PD 168393 has been shown to increase neurite growth *in vitro* (e.g., Refs 44 and 45). Query results from RegenBase identify EGFR as well as ERBB proteins as strongly inhibited targets of PD 168393. However, knocking down EGFR does not promote neurite growth, suggesting that PD 168393 acts on targets other than (or in addition to) neuronal EGFRs, either EGFRs on other cell types or other molecular targets on neurons (46). ERBB2 has no ligand binding domain of its own but forms heterocomplexes with other ERBB family members; ERBB4 binds neuregulins and its activation positively regulates neurite growth (45). This suggests that PD 168393’s ability to improve BBB score is not owing to its inhibition of EGFRs/ERBBs on neurons; perhaps, the relevant effects are on glial receptors.

Finally, SB 203580, typically reported as a p38 kinase inhibitor, inhibits a large number of kinase targets, many of which are relevant to axon growth. Improvement in behavioral outcomes following SCI in animals treated with SB 203580 has been attributed to p38 kinase inhibition (47). However, our recent work implies that both p38-alpha (mitogen-activated kinase MAPK14) and p38-beta (MAPK11) are ‘anti-targets’, whose inhibition should be avoided in order to promote axon growth in hippocampal neurons (31). This conclusion is in line with previous suggestions that p38s are positive regulators of neurite growth, and that inhibition of p38 activity suppresses growth promotion through Nerve Growth Factor (48–50). Results of our queries to RegenBase suggest an alternative mechanism for SB 203580: it inhibits both JNK3 and Casein Kinase 1 (CSNK1A1, CSNK1D and CSNK1E), which are negative regulators of axon growth. This suggests that inhibition of JNK3 and CSNK1s by SB 203580 enhances axon growth. Perhaps, therefore, JNK3 and the CSNK1s are the targets of SB 203580 mediating the reported positive behavioral outcomes *in vivo*. An alternative explanation is that SB 203580 exerts its *in vivo* effect by acting on cells other than neurons (see Ref. 31).

The above query illustrates how RegenBase enables the progression of inquiry from a general question about *in vivo* studies and high throughput screening data to specific hypotheses about novel targets and compounds that could be tested *in vitro* or *in vivo*. For example, does knocking out or knocking down PRKX in neurons *in vivo* enhance axon regeneration, or improve behavioral outcomes after CNS injury? The literature offers conflicting information about the role of p38/MAPK in axon regeneration. SB 203580 is used as a p38 (MAPK11 and MAP14) inhibitor and this is reported to enhance optic nerve regeneration (51). In contrast, MAPK14 loss of function leads to reduced axon regeneration in a sciatic nerve crush model (52). It seems likely that SB 203580 promotes optic nerve regeneration by acting either on a non-p38 target in retinal ganglion cells (RGCs) or on p38s in other cell types such as Müller cells or astrocytes, to help RGCs survive or regenerate. RegenBase provides a ‘tool for thought’ to arrive at these novel biological hypotheses (53), which we plan to test experimentally.

### Querying RegenBase to identify drugs of interest

In this section, we present two additional examples of using RegenBase to identify perturbagens of interest to the SCI research community. RegenBase allows researchers to aggregate experimental findings across published studies, and to use the RB class hierarchy to identify related behavioral outcome measures widely used to assess functional recovery following SCI in mouse and rat models.

### QUERY 2—What perturbagens have been observed to improve behavioral outcomes following injury?

QUERY 2 retrieves all events where the target is of type ‘outcome measure’ or any of its subclasses, and where the agent is of type ‘small molecule’ and has an ‘increase’ effect on the target. It returns the distinct set of compound identifiers of the agent. The SPARQL query is shown in Supplementary Appendix S3; Table 2 shows the compounds returned by this query with their names.

**Table 2.** The identifiers and names of 18 compounds that have been experimentally observed to improve behavioral outcomes following SCI

PubChem identifiers.org identifier	Name
http://identifiers.org/pubchem.compound/5957	Adenosine triphosphate
http://identifiers.org/pubchem.compound/2046	AG 126
http://identifiers.org/pubchem.compound/5328775	AG 556
http://identifiers.org/pubchem.compound/72941992	AM 111
http://identifiers.org/pubchem.compound/9813758	BMS 345541
http://identifiers.org/pubchem.compound/10224714	CR8
http://identifiers.org/pubchem.compound/3547	Fasudil
http://identifiers.org/pubchem.compound/6324617	Ginkgolide B
http://identifiers.org/pubchem.compound/3973	LY 294002
http://identifiers.org/pubchem.compound/6741	Methylpre-dnisolone
http://identifiers.org/pubchem.compound/4708	PD 168393
http://identifiers.org/pubchem.compound/4713	PD 98059
http://identifiers.org/pubchem.compound/126402	PRE 084
http://identifiers.org/pubchem.compound/5040	Rapamycin
http://identifiers.org/pubchem.compound/176155	SB 203580
http://identifiers.org/pubchem.compound/4210951	SB 415286
http://identifiers.org/pubchem.compound/4124851	TDZD-8
http://identifiers.org/pubchem.compound/3006531	U0126

QUERY 2 leverages the class hierarchy of RB to ask for data about any behavioral outcome (entities that are instances of any subclass of the RB class ‘behavioral outcome’, RB_0008016). This kind of query allows one to quickly identify treatments that could be used in combination to look for synergistic effects of compounds *in vitro* or *in vivo*.

### QUERY 3—What perturbagens have been observed to improve BBB or BMS score following injury and also to increase neurite outgrowth? How many assay results report the neurite outgrowth effect? How many articles report the BBB/BMS score effect?

QUERY 3 retrieves all events where the agent is a small molecule and the target is a BBB or BMS score, where the curated source article reported that the agent increased BBB or BMS score. An additional constraint is that the agent must also have been observed to increase neurite outgrowth relative to a control, in at least one separate assay (of type ‘neurite outgrowth assay’, from the BAO). The query returns the identifiers of all agents that satisfy these constraints as well as the number of assay results reporting the effect on neurite growth and the number of articles reporting the effect on BBB/BMS score. The SPARQL query is shown in Supplementary Appendix S3; query results are shown in Table 3.

**Table 3.** Compounds that have been experimentally observed to improve BBB or BMS score following injury and which have independently been observed to increase neurite outgrowth

PubChem identifiers.org identifier	Name	Number of assays	Number of articles
http://identifiers.org/pubchem.compound/10224714	CR8	2	2
http://identifiers.org/pubchem.compound/3547	Fasudil	1	4

A large variety of treatments are tested *in vivo* for improving various behavioral outcomes after SCI. For example, some treatments, such as the CDK inhibitor CR8, are hypothesized to improve pain management by reducing microglial activation. This search result indicates that the treatment could also be directly influencing neurons, perhaps by altering sprouting after injury. Therefore, RegenBase can easily allow the generation of new hypotheses about cellular targets for various treatments that can be subsequently investigated.

## Discussion

To our knowledge, RegenBase is the first publicly available resource that structures and automatically integrates SCI domain knowledge with literature-sourced and raw experimental data. This work marks an important step forward in applying informatics tools and methods to drive discoveries in SCI research. With its current focus on regeneration, RegenBase enables the formulation of hypotheses about potential drug targets, and how they may improve recovery after SCI.

Although we were successful in the technical exercise of integrating literature sourced reports of experimental observations with relevant assay data, and structuring this information with a domain ontology to facilitate querying, our approach has several limitations. One of the primary challenges in developing RegenBase is the technical knowledge required to develop and implement data representation schemes, as well as to use query languages such as SPARQL for interrogation. The technical expertise required for developing RegenBase is not unique to this project—all biomedical databases rely on expert architects and administrators to create and maintain them over time. As the adoption of Semantic Web standards for structuring and querying data on the web continues to increase, so too does the availability of resources designed to instruct and aid both the experienced developer and the new user (e.g. Ref. 54). Recent work has also focused on developing user-friendly tools for constructing SPARQL queries that auto-suggest types and predicates to lower the barrier to use, e.g. Ref. 55, or that allow users to define queries using a controlled natural language which are then converted to SPARQL on the fly, e.g. Ref. 56. Front-end search interfaces that provide an entry point to biochemical data structured using ontologies, such as the LINCS Information Framework (LIFE) project (57) (see http://life.ccs.miami.edu/life/), also make resources like RegenBase accessible to a broader user base.

The manual effort and domain expertise required for literature curation is another limitation in developing resources such as RegenBase. Our curators estimate that curating one publication requires between 1 and 3 h. We are mitigating this by developing an online tool for literature curation using MIASCI as its backbone, which is publicly available at http://regenbase.org/miasci-online.html. Our aim is to streamline the process for SCI researchers to curate their own research as it is published, and also to curate previously published work. Assessing the scalability of our curation and data collection approach as well as the effect of inter-curator variability is a multi-year effort, but one that will be possible as curators and users contibute data to RegenBase over time.

Crowdsourcing approaches have also shown great promise in reducing the overhead of manual curation. Crowdsourcing biocuration tasks via online platforms such as Amazon’s Mechanical Turk (where workers are paid for completing tasks) produces both accurate and comprehensive results (58, 59). Crowdsourcing curation of abstracts to identify a limited set of SCI experimental metadata such as the animal models, injury types and perturbagens studied as well as primary observed outcomes would be an exciting potential advance for RegenBase. Our hypothesis is that the sheer scale of such crowdsourced curated data will outweigh potential inaccuracies introduced by the variation in worker ability and domain knowledge. However, selecting successful tasks and incentives for crowdsourcing is a research area in itself, and far from a solved problem. The success of the endeavor will also depend on the quality of available abstracts, and we believe that initially such crowdsourced data should augment, but not replace, a smaller but richer knowledge base constructed by experts curating entire published research papers.

Crowdsourcing approaches to populate RegenBase would benefit greatly from community incentives to reward the effort of curating literature and making experimental data publicly available. A data publishing platform allowing scientists to get credit for sharing their data (including negative results) and also to cite data they use in their research or contribute to resources like RegenBase would offer just such an incentive. A system by which to track scientific contribution by data publishing would also advance the scientific community as a whole by encouraging researchers to make their data publicly available and by making the collection of ‘altmetrics’ for scientific contribution (such as dataset downloads and reuse) possible. Newer forms of publication venues, such as the journal Nature Scientific Data, make such an incentive scheme feasible.

The data representations we have developed to structure reported experimental results for RegenBase offer several advantages. Firstly, they capture different types of experimental data from a variety of sources in a consistent manner, making query formulation easier. For example, a query to retrieve kinase percent inhibition screening data needs to be modified only slightly to retrieve kinase p*K*_d_/p*K*_i_ data. As RegenBase continues to grow (we are continually adding literature-sourced statements and experimental data), it will be increasingly important to be able to reuse our existing data representations (and the code that implements them) to maintain a scalable workflow.

Secondly, our use of a consistent identifier scheme when creating linked data from different sources enables automatic linking of assay experimental results to each other and to literature-sourced data, as demonstrated by the queries presented above. Our use of identifiers.org URIs automatically links RegenBase statements to the larger public linked data network of biological and biochemical resources which provide additional information about the genes, proteins, and chemicals in RegenBase (60–63). This offers the potential to perform analyses using RegenBase alongside additional sources, such as information about protein family relationships, other biochemical profiling data, GO annotations, and model organism specific databases such as the Mouse Genome Informatics database (64) and Rat Genome Database (65, 66) to make additional discoveries and develop new hypotheses for experimental testing.

Finally, the data representations we developed consider all reported events and assay results as contextually true within the scope of the source publication or dataset, but purposely make no claims as to their global truth. One of our aims for RegenBase is to allow its users to identify contradictory facts and data across publications, a task that if undertaken manually would require hours of reading and data integration. By allowing potentially contradictory data to coexist in RegenBase, a simple query allows users to identify such cases. In future work, we will explore methods for automatically identifying contradictory data, as well as approaches for displaying or flagging such data for users.

Domains that are closely related to SCI, such as traumatic brain injury (TBI) and optic nerve regeneration, would also benefit from RegenBase’s structured representation of experimental data and literature findings. Efforts to develop Common Data Elements for describing preclinical TBI experiments (67) mirror our work on RegenBase and set the stage for extending RegenBase to the domain of TBI. The high prevalence of glaucoma and resulting optic nerve injuries has inspired a significant effort to identify gene targets and therapeutics to promote optic nerve regeneration, e.g. Refs 68 and 69. Importantly, there is also a significant overlap in protein targets such as phosphatase and tensin homolog (70, 71) and Krüppel-like factors (72, 73) in optic nerve and spinal cord axon regeneration. The ability to query a knowledge base across these disparate domains could speed hypothesis generation and testing in both areas.

In conclusion, we have developed RegenBase, a knowledge base for SCI biology. We have demonstrated its use to answer domain-specific questions and develop biological hypotheses by computationally aggregating facts and data from multiple sources. RegenBase is publicly available at http://regenbase.org. Its use by the scientific community and resulting feedback will allow us to continue to develop and extend the functionality of this resource.

## Supplementary data

Supplementary data are available at *Database* Online.

Supplementary Data
