# Supplementary material for: RegenBase: a knowledge base of spinal cord injury biology for translational research
Source: Database (Oxford). 2016 Apr 6;2016:baw040. doi: 10.1093/database/baw040 (PMC4823819; doi:10.1093/database/baw040)
Supplement: Supplementary Data [file supp_2016_baw040_index.html]

Supplementary Data 

# RegenBase: a knowledge base of spinal cord injury biology for translational research

## Supplementary Data

files

- Supplementary Data - docx file
