# Supplementary material for: RegenBase: a knowledge base of spinal cord injury biology for translational research
Source: Database (Oxford). 2016 Apr 6;2016:baw040. doi: 10.1093/database/baw040 (PMC4823819; doi:10.1093/database/baw040)
Supplement: Supplementary Data [file supp_baw040_Callahan_et_al_RegenBase_revision_final.docx]

## Appendix 1 – PubMed search terms to retrieve publications that were mined for RegenBase ontology seed concepts

Axon growth

Gliosis and Spinal Cord Injury

Growth cone attraction

Growth cone collapse

Growth cone guidance

Growth cone turning assay

miRNA and Spinal Cord Injury

Neurite outgrowth assay

Neurite extension

Neuroprotection and Spinal Cord Injury

Spinal cord and Microarray

NMDA and Spinal Cord Injury

Radiation and Spinal Cord Injury

Inflammation and Spinal Cord Injury

Spinal Cord Contusion Injury

Astrocyte and Spinal Cord Injury

Apoptosis and Spinal Cord Injury

Autophagy and Spinal Cord Injury

Cell death and Spinal Cord Injury

Chemical injury and Spinal Cord Injury

Macrophages and Spinal Cord Injury

Neuropathic Pain and Spinal Cord Injury

Oligodendrocyte and Spinal Cord Injury

Ischemic injury and Spinal Cord Injury

Sprouting and Spinal Cord Injury

Recovery and Spinal Cord Injury

## Appendix 2 – Manually curated publications in RegenBase

Cuzzocrea S, Genovese T, Mazzon E, Crisafulli C, Di Paola R, Muia C, Collin M, Esposito E, Bramanti P, Thiemermann C: **Glycogen synthase kinase-3 beta inhibition reduces secondary damage in experimental spinal cord trauma**. *J Pharmacol Exp Ther* 2006, **318**(1):79-89.

Nishio Y, Koda M, Kitajo K, Seto M, Hata K, Taniguchi J, Moriya H, Fujitani M, Kubo T, Yamashita T: **Delayed treatment with Rho-kinase inhibitor does not enhance axonal regeneration or functional recovery after spinal cord injury in rats**. *Exp Neurol* 2006, **200**(2):392-397.

Yamauchi K, Osuka K, Takayasu M, Usuda N, Nakazawa A, Nakahara N, Yoshida M, Aoshima C, Hara M, Yoshida J: **Activation of JAK/STAT signalling in neurons following spinal cord injury in mice**. *J Neurochem* 2006, **96**(4):1060-1070.

Xu Z, Wang BR, Wang X, Kuang F, Duan XL, Jiao XY, Ju G: **ERK1/2 and p38 mitogen-activated protein kinase mediate iNOS-induced spinal neuron degeneration after acute traumatic spinal cord injury**. *Life Sci* 2006, **79**(20):1895-1905.

Fabes J, Anderson P, Brennan C, Bolsover S: **Regeneration-enhancing effects of EphA4 blocking peptide following corticospinal tract injury in adult rat spinal cord**. *Eur J Neurosci* 2007, **26**(9):2496-2505.

Genovese T, Mazzon E, Esposito E, Muia C, Di Paola R, Crisafulli C, Bramanti P, Cuzzocrea S: **Inhibition of tyrosine kinase-mediated cellular signalling by Tyrphostins AG126 and AG556 modulates secondary damage in experimental spinal cord trauma**. *Neuropharmacology* 2007, **52**(7):1454-1471.

Zhao P, Waxman SG, Hains BC: **Extracellular signal-regulated kinase-regulated microglia-neuron signaling by prostaglandin E2 contributes to pain after spinal cord injury**. *J Neurosci* 2007, **27**(9):2357-2368.

Dill J, Wang H, Zhou F, Li S: **Inactivation of glycogen synthase kinase 3 promotes axonal growth and recovery in the CNS**. *J Neurosci* 2008, **28**(36):8914-8928.

Genovese T, Esposito E, Mazzon E, Muia C, Di Paola R, Meli R, Bramanti P, Cuzzocrea S: **Evidence for the role of mitogen-activated protein kinase signaling pathways in the development of spinal cord injury**. *J Pharmacol Exp Ther* 2008, **325**(1):100-114.

Kajana S, Goshgarian HG: **Spinal activation of the cAMP-PKA pathway induces respiratory motor recovery following high cervical spinal cord injury**. *Brain Res* 2008, **1232**:206-213.

Stirling DP, Liu J, Plunet W, Steeves JD, Tetzlaff W: **SB203580, a p38 mitogen-activated protein kinase inhibitor, fails to improve functional outcome following a moderate spinal cord injury in rat**. *Neuroscience* 2008, **155**(1):128-137.

Furuya T, Hashimoto M, Koda M, Okawa A, Murata A, Takahashi K, Yamashita T, Yamazaki M: **Treatment of rat spinal cord injury with a Rho-kinase inhibitor and bone marrow stromal cell transplantation**. *Brain Res* 2009, **1295**:192-202.

Gwak YS, Unabia GC, Hulsebosch CE: **Activation of p-38alpha MAPK contributes to neuronal hyperexcitability in caudal regions remote from spinal cord injury**. *Exp Neurol* 2009, **220**(1):154-161.

Chiba Y, Kuroda S, Shichinohe H, Hokari M, Osanai T, Maruichi K, Yano S, Hida K, Iwasaki Y: **Synergistic effects of bone marrow stromal cells and a Rho kinase (ROCK) inhibitor, fasudil on axon regeneration in rat spinal cord injury**. *Neuropathology* 2010, **30**(3):241-250.

Hu LY, Sun ZG, Wen YM, Cheng GZ, Wang SL, Zhao HB, Zhang XR: **ATP-mediated protein kinase B Akt/mammalian target of rapamycin mTOR/p70 ribosomal S6 protein p70S6 kinase signaling pathway activation promotes improvement of locomotor function after spinal cord injury in rats**. *Neuroscience* 2010, **169**(3):1046-1062.

Han M, Huang RY, Du YM, Zhao ZQ, Zhang YQ: **Early intervention of ERK activation in the spinal cord can block initiation of peripheral nerve injury-induced neuropathic pain in rats**. *Sheng Li Xue Bao* 2011, **63**(2):106-114.

Paterniti I, Esposito E, Mazzon E, Bramanti P, Cuzzocrea S: **Evidence for the role of PI(3) -kinase-AKT-eNOS signalling pathway in secondary inflammatory process after spinal cord compression injury in mice**. *Eur J Neurosci* 2011, **33**(8):1411-1420.

Renault-Mihara F, Katoh H, Ikegami T, Iwanami A, Mukaino M, Yasuda A, Nori S, Mabuchi Y, Tada H, Shibata S *et al*: **Beneficial compaction of spinal cord lesion by migrating astrocytes through glycogen synthase kinase-3 inhibition**. *EMBO Mol Med* 2011, **3**(11):682-696.

Erschbamer M, Pernold K, Olson L: **Comments on the re-assessment study by Sharp et al. of Erschbamer et al**. *Exp Neurol* 2012, **233**(2):660-661.

Han X, Lu M, Wang S, Lv D, Liu H: **Targeting IKK/NF-kappaB pathway reduces infiltration of inflammatory cells and apoptosis after spinal cord injury in rats**. *Neurosci Lett* 2012, **511**(1):28-32.

Impellizzeri D, Mazzon E, Paterniti I, Esposito E, Cuzzocrea S: **Effect of fasudil, a selective inhibitor of Rho kinase activity, in the secondary injury associated with the experimental model of spinal cord trauma**. *J Pharmacol Exp Ther* 2012, **343**(1):21-33.

Li S, He J, Li S, Cao G, Tang S, Tong Q, Joshi HC: **Noscapine induced apoptosis via downregulation of survivin in human neuroblastoma cells having wild type or null p53**. *PloS one* 2012, **7**(7):e40076.

Qu WS, Tian DS, Guo ZB, Fang J, Zhang Q, Yu ZY, Xie MJ, Zhang HQ, Lu JG, Wang W: **Inhibition of EGFR/MAPK signaling reduces microglial inflammatory response and the associated secondary damage in rats after spinal cord injury**. *J Neuroinflammation* 2012, **9**:178.

Repici M, Chen X, Morel MP, Doulazmi M, Sclip A, Cannaya V, Veglianese P, Kraftsik R, Mariani J, Borsello T *et al*: **Specific inhibition of the JNK pathway promotes locomotor recovery and neuroprotection after mouse spinal cord injury**. *Neurobiol Dis* 2012, **46**(3):710-721.

Sekiguchi A, Kanno H, Ozawa H, Yamaya S, Itoi E: **Rapamycin promotes autophagy and reduces neural tissue damage and locomotor impairment after spinal cord injury in mice**. *Journal of neurotrauma* 2012, **29**(5):946-956.

Sharp K, Yee KM, Steward O: **A re-assessment of the effects of treatment with an epidermal growth factor receptor (EGFR) inhibitor on recovery of bladder and locomotor function following thoracic spinal cord injury in rats**. *Exp Neurol* 2012, **233**(2):649-659.

Tan YH, Li K, Chen XY, Cao Y, Light AR, Fu KY: **Activation of Src family kinases in spinal microglia contributes to formalin-induced persistent pain state through p38 pathway**. *J Pain* 2012, **13**(10):1008-1015.

Wu J, Stoica BA, Dinizo M, Pajoohesh-Ganji A, Piao C, Faden AI: **Delayed cell cycle pathway modulation facilitates recovery after spinal cord injury**. *Cell Cycle* 2012, **11**(9):1782-1795.

Wu J, Pajoohesh-Ganji A, Stoica BA, Dinizo M, Guanciale K, Faden AI: **Delayed expression of cell cycle proteins contributes to astroglial scar formation and chronic inflammation after rat spinal cord contusion**. *J Neuroinflammation* 2012, **9**:169.

Berta T, Liu YC, Xu ZZ, Ji RR: **Tissue plasminogen activator contributes to morphine tolerance and induces mechanical allodynia via astrocytic IL-1beta and ERK signaling in the spinal cord of mice**. *Neuroscience* 2013, **247**:376-385.

Gwak YS, Hassler SE, Hulsebosch CE: **Reactive oxygen species contribute to neuropathic pain and locomotor dysfunction via activation of CamKII in remote segments following spinal cord contusion injury in rats**. *Pain* 2013, **154**(9):1699-1708.

Lee JY, Choi DC, Oh TH, Yune TY: **Analgesic effect of acupuncture is mediated via inhibition of JNK activation in astrocytes after spinal cord injury**. *PloS one* 2013, **8**(9):e73948.

Moon JY, Roh DH, Yoon SY, Kang SY, Choi SR, Kwon SG, Choi HS, Han HJ, Beitz AJ, Lee JH: **Sigma-1 receptor-mediated increase in spinal p38 MAPK phosphorylation leads to the induction of mechanical allodynia in mice and neuropathic rats**. *Exp Neurol* 2013, **247**:383-391.

Seo TB, Chang IA, Lee JH, Namgung U: **Beneficial function of cell division cycle 2 activity in astrocytes on axonal regeneration after spinal cord injury**. *Journal of neurotrauma* 2013, **30**(12):1053-1061.

Song Y, Liu J, Zhang F, Zhang J, Shi T, Zeng Z: **Antioxidant effect of quercetin against acute spinal cord injury in rats and its correlation with the p38MAPK/iNOS signaling pathway**. *Life Sci* 2013, **92**(24-26):1215-1221.

Song Y, Zeng Z, Jin C, Zhang J, Ding B, Zhang F: **Protective effect of ginkgolide B against acute spinal cord injury in rats and its correlation with the Jak/STAT signaling pathway**. *Neurochem Res* 2013, **38**(3):610-619.

Sun Z, Hu L, Wen Y, Chen K, Sun Z, Yue H, Zhang C: **Adenosine triphosphate promotes locomotor recovery after spinal cord injury by activating mammalian target of rapamycin pathway in rats**. *Neural Regen Res* 2013, **8**(2):101-110.

Wu J, Raver C, Piao C, Keller A, Faden AI: **Cell cycle activation contributes to increased neuronal activity in the posterior thalamic nucleus and associated chronic hyperesthesia after rat spinal cord contusion**. *Neurotherapeutics* 2013, **10**(3):520-538.

Wu BQ, Bi ZG, Qi Q: **Inactivation of the Rho-ROCK signaling pathway to promote neurologic recovery after spinal cord injuries in rats**. *Chin Med J (Engl)* 2013, **126**(19):3723-3727.

He BR, Xie ST, Wu MM, Hao DJ, Yang H: **Phagocytic removal of neuronal debris by olfactory ensheathing cells enhances neuronal survival and neurite outgrowth via p38MAPK activity**. *Mol Neurobiol* 2014, **49**(3):1501-1512.

Li ZW, Li JJ, Wang L, Zhang JP, Wu JJ, Mao XQ, Shi GF, Wang Q, Wang F, Zou J: **Epidermal growth factor receptor inhibitor ameliorates excessive astrogliosis and improves the regeneration microenvironment and functional recovery in adult rats following spinal cord injury**. *J Neuroinflammation* 2014, **11**:71.

Wang X, Hu J, She Y, Smith GM, Xu XM: **Cortical PKC inhibition promotes axonal regeneration of the corticospinal tract and forelimb functional recovery after cervical dorsal spinal hemisection in adult rats**. *Cereb Cortex* 2014, **24**(11):3069-3079.

## Appendix 3 – Queries to RegenBase

Below are 5 SPARQL queries that can be used to retrieve data from RegenBase. The results of queries 1 -3 are presented in the main body of the manuscript. We provide results of queries 4 and 5 below – these queries offer a broader view by retrieving the injury and outcome types instantiated in RegenBase.

**QUERY 1 – What are the kinase targets of perturbagens that have been observed to increase BBB/BMS score and inhibit kinase activity with either a % inhibition of at least 50% at a perturbagen concentration of 0.1 μM or a pK_d/_pK_i_ of at least 7?**

PREFIX bao: <http://www.bioassayontology.org/bao#>

PREFIX bibtex: <http://purl.org/net/nknouf/ns/bibtex#>

PREFIX hyque: <http://semanticscience.org/ontology/hyque.owl#>

PREFIX regenbase: <http://regenbase.org/ontology#>

PREFIX sio: <http://semanticscience.org/resource/>

PREFIX uo: <http://purl.org/obo/owl/UO#>

# return distinct compound and protein identifier pairs

SELECT DISTINCT ?pubchem_id ?uniprot_id

WHERE {

# retrieve event, agent, target and effect

?event hyque:HYPOTHESIS_0000015 ?agent .

?event bibtex:hasKey ?article .

?agent sio:SIO_000671 ?identifier .

?event hyque:HYPOTHESIS_0000016 ?target .

# filter agent to be a small molecule

?agent rdf:type regenbase:RB_0000125 .

?agent ?effect ?target .

?effect rdfs:label ?effect_label .

# filter effect to be only “increase”

FILTER(?effect_label = "increase")

# filter target to be a BBB or BMS score

{ ?target rdf:type regenbase:RB_0008017 }

UNION { ?target rdf:type regenbase:RB_0008018 }

UNION { ?target rdf:type regenbase:RB_0008019 }

UNION { ?target rdf:type regenbase:RB_0008020 }.

BIND(IRI(?identifier) AS ?pubchem_id)

{

# filter assay to be a kinase activity assay

?assay rdf:type bao:BAO_0002005 .

?compound bao:BAO_0000383 ?pubchem_id .

?assay bao:BAO_0000185 ?compound .

?assay bao:BAO_0000209 ?measuregroup .

?measuregroup bao:BAO_0000208 ?endpoint .

# filter endpoint to be a ‘percent response’

?endpoint rdf:type bao:BAO_0001103 .

?endpoint bao:BAO_0000195 ?value .

?endpoint bao:BAO_0000338 ?conc .

?conc bao:BAO_0000183 ?conc_unit .

# filter concentration unit to be micromolar

?conc_unit rdf:type uo:UO_0000064 .

?conc bao:BAO_0002666 ?conc_value .

# filter concentration value to be 0.1 micromolar

FILTER (?conc_value = 0.1) .

# filter endpoint value to be at or above threshold of 50

FILTER(?value >= 50).

# retrieve assay target

?measuregroup bao:BAO_0000211 ?kinase_target .

?kinase_target bao:BAO_0000383 ?uniprot_id .

} UNION {

# filter assay to be a ‘selectivity assay’

?assay rdf:type bao:BAO_0000478 .

?compound bao:BAO_0000383 ?pubchem_id .

?assay bao:BAO_0000185 ?compound .

?assay bao:BAO_0000209 ?measuregroup .

?measuregroup bao:BAO_0000208 ?endpoint .

# filter endpoint to be a ‘binding constant’

?endpoint rdf:type bao:BAO_0000587 .

?endpoint bao:BAO_0095007 ?value .

# filter endpoint value to be at or above threshold of 7

FILTER(?value >=7)

# retrieve assay target

?measuregroup bao:BAO_0000211 ?kinase_target .

?kinase_target bao:BAO_0000383 ?uniprot_id .

} UNION {

# filter assay to be a ‘selectivity assay’

?assay rdf:type bao:BAO_0000478 .

?compound bao:BAO_0000383 ?pubchem_id .

?assay bao:BAO_0000185 ?compound .

?assay bao:BAO_0000209 ?measuregroup .

?measuregroup bao:BAO_0000208 ?endpoint .

# filter endpoint to be a ‘computational endpoint’

?endpoint rdf:type bao:BAO_0002137 .

?endpoint bao:BAO_0095000 ?endpoint2 .

?endpoint2 rdf:type bao:BAO_0002137 .

?endpoint bao:BAO_0095007 ?value .

# filter endpoint value to be at or above threshold of 7

FILTER(?value >=7)

# retrieve assay target

?measuregroup bao:BAO_0000211 ?kinase_target .

?kinase_target bao:BAO_0000383 ?uniprot_id .

}

} ORDER BY ?pubchem_id ?uniprot_id

**QUERY 2 – What perturbagens have been observed to improve behavioral outcomes following injury?**

PREFIX hyque: <http://semanticscience.org/ontology/hyque.owl#>

PREFIX regenbase: <http://regenbase.org/ontology#>

PREFIX sio: <http://semanticscience.org/resource/>

# return distinct agent PubChem compound identifiers

SELECT DISTINCT ?pubchem_id

WHERE {

# retrieve agent, target, and effect

?event hyque:HYPOTHESIS_0000015 ?agent .

?agent sio:SIO_000671 ?pubchem_id .

?event hyque:HYPOTHESIS_0000016 ?target .

# filter agent to be a small molecule

?agent rdf:type regenbase:RB_0000125 .

?agent ?effect ?target .

?effect rdfs:label ?effect_label .

# filter effect to be ‘increase’

FILTER(?effect_label = "increase") .

# filter target to be an outcome measure or any subclass

?target rdf:type/rdfs:subClassOf* regenbase:RB_0008016 .

}

**QUERY 3 – What perturbagens have been observed to improve BBB or BMS score following injury and also to increase neurite outgrowth? How many assay results report the neurite outgrowth effect? How many articles report the BBB/BMS score effect?**

PREFIX bao: <http://www.bioassayontology.org/bao#>

PREFIX bibtex: <http://purl.org/net/nknouf/ns/bibtex#>

PREFIX hyque: <http://semanticscience.org/ontology/hyque.owl#>

PREFIX regenbase: <http://regenbase.org/ontology#>

PREFIX sio: <http://semanticscience.org/resource/>

SELECT DISTINCT ?pubchem_id COUNT(DISTINCT ?assay) AS ?num_assays COUNT(DISTINCT ?article) AS ?num_articles

WHERE {

#retrieve agent, target and effect

?event hyque:HYPOTHESIS_0000015 ?agent .

?event bibtex:hasKey ?article .

?agent sio:SIO_000671 ?identifier .

?event hyque:HYPOTHESIS_0000016 ?target .

# filter agent to be a small molecule

?agent rdf:type regenbase:RB_0000125 .

?agent ?effect ?target .

?effect rdfs:label ?effect_label .

# filter effect to be ‘increase’

FILTER(?effect_label = "increase")

# filter target to be a BBB or BMS score

{ ?target rdf:type regenbase:RB_0008017 }

UNION { ?target rdf:type regenbase:RB_0008018 }

UNION { ?target rdf:type regenbase:RB_0008019 }

UNION { ?target rdf:type regenbase:RB_0008020 }.

BIND(IRI(?identifier) AS ?pubchem_id).

?compound bao:BAO_0000383 ?pubchem_id .

?assay bao:BAO_0000185 ?compound .

?assay rdf:type bao:BAO_0002803.

?assay bao:BAO_0000208 ?endpoint .

?endpoint bao:BAO_0000195 ?value .

# filter percent neurite outgrowth to be 120% or more

FILTER(?value >= 120)

}

**QUERY 4: What is the distribution of injury types in RegenBase?**

This query retrieves all events where the agent is of type injury or any of its subclasses, and returns the number of articles that are the source of annotated events for each injury type. Table 4 shows the results of this query. Spinal cord injuries in general have the highest number of associated articles, followed by contusive injuries. Articles for which a curator has indicated a more specific type of spinal cord injury (for example, an Infinite Horizon impactor injury) are fewer in number.

PREFIX bibtex: <http://purl.org/net/nknouf/ns/bibtex#>

PREFIX hyque: <http://semanticscience.org/ontology/hyque.owl#>

PREFIX regenbase: <http://regenbase.org/ontology#>

PREFIX sio: <http://semanticscience.org/resource/>

SELECT DISTINCT STR(?injury_type) AS ?injury_type COUNT(DISTINCT ?article) AS ?num_articles

WHERE {

?event hyque:HYPOTHESIS_0000015 ?agent .

?event bibtex:hasKey ?article .

?agent rdf:type/rdfs:subClassOf* ?agent_type .

?agent_type rdfs:label ?injury_type .

?agent_type rdfs:subClassOf* regenbase:RB_0000287 .

} ORDER BY DESC(?num_articles)

Table 4 The injury types in RegenBase and the number of articles with an annotation describing an experiment involving an injury of a given type.

| **Injury type** | **Number of articles** |
| --- | --- |
| injury | 19 |
| nervous system injury | 19 |
| spinal cord injury | 19 |
| contusive injury | 12 |
| spinal cord contusive injury | 12 |
| weight drop injury | 6 |
| impactor injury | 3 |
| transection | 2 |
| spinal cord transection | 2 |
| clip compression injury | 2 |
| Infinite Horizon impactor injury | 1 |
| Ohio State University impactor injury | 1 |
| NYU impactor injury | 1 |
| incomplete transection | 1 |
| dorsal hemisection | 1 |

**QUERY 5: What is the distribution of observed outcomes in RegenBase?**

This query retrieves the types for all targets in the RegenBase, and the number of articles associated with each type. There is a total of 77 target types in the RegenBase KB. For brevity, Table 5 shows a subset of these types, ordered by the number of articles that sourced an annotation about the type.

PREFIX bibtex: <http://purl.org/net/nknouf/ns/bibtex#>

PREFIX hyque: <http://semanticscience.org/ontology/hyque.owl#>

PREFIX regenbase: <http://regenbase.org/ontology#>

PREFIX sio: <http://semanticscience.org/resource/>

SELECT DISTINCT STR(?target_label) AS ?target_label COUNT(DISTINCT ?article) AS ?num_articles

WHERE {

?event bibtex:hasKey ?article .

?event hyque:HYPOTHESIS_0000016 ?target .

?target rdf:type/rdfs:subClassOf* ?target_type .

?target_type rdfs:label ?target_label .

} ORDER BY DESC(?num_articles)

Table 5 15 of the 77 target types in RegenBase KB and the number of articles with an annotation about a given target type.

| **Target type** | **Number of articles** |
| --- | --- |
| behavioral assessment | 28 |
| BBB score | 21 |
| phosphorylation | 21 |
| enzyme | 15 |
| serine-threonine kinase | 10 |
| biological regulation | 10 |
| positive regulation of gene expression | 9 |
| allodynia | 6 |
| enzyme activity | 6 |
| positive regulation of phosphorylation | 5 |
| BMS score | 5 |
| NeuN positive cells | 3 |
| lesion volume | 3 |
| decreased thermal nociceptive threshold | 3 |
| ischuria | 3 |
